# Emodin promotes GSK-3β-mediated PD-L1 proteasomal degradation and enhances anti-tumor immunity in hepatocellular carcinoma

**DOI:** 10.1186/s13020-025-01146-6

**Published:** 2025-08-13

**Authors:** Xuemei Yang, Weiguang Chen, Haitao Sun, Weicong Chen, Wei Xu, Chunyu He, Yang Liu, Ying Kuang, Yanhao Ma, Binglian Zhong, Chaojie Li, Guohuan Li, Qingfeng Du, Songqi He

**Affiliations:** 1 Affiliated Zhuhai Hospital, Southern Medical University (Zhuhai Hospital of Integrated Traditional Chinese and Western Medicine), Zhuhai, 519000 China; 2 School of Traditional Chinese Medicine, Southern Medical University, Guangzhou, 510515 China; 3 The People’s Hospital Medical Group of Xiangzhou, Zhuhai, 519099 China

**Keywords:** Hepatocellular carcinoma, Emodin, PD-L1, GSK-3β

## Abstract

**Background:**

Programmed death-ligand 1 (PD-L1), a prominent immune checkpoint, interacts with programmed death protein-1 (PD-1) on cytotoxic T cells within tumors and promotes immune evasion. Emodin, which is known to destabilize PD-L1 in breast cancer, has great potential for enhancing anti-tumor immunity. However, whether emodin can modulate PD-L1 levels in hepatocellular carcinoma (HCC) and enhance anti-tumor immune response remains unclear.

**Materials and methods:**

PD-L1 levels were assessed by western blot and RT-qPCR, the degradation mechanism was analyzed using specific inhibitors. Network pharmacology, molecular docking, and glycogen synthase kinase-3 beta (GSK-3β) modulation analyzes were performed to validate emodin’s target. In vivo anti-tumor effects were evaluated in H_22_ subcutaneous tumor model, and CD8^+^ T cells and RNA-seq data were analyzed. The synergistic effects of emodin and an anti-PD-L1 antibody were assessed.

**Results:**

Emodin effectively reduced PD-L1 levels in H_22_ cells and increased anti-tumor activity in an H_22_ subcutaneous tumor model by promoting CD8^+^ T cells infiltration and TNF-α, IFN-γ, and granzyme B secretion. Mechanistically, emodin accelerated PD-L1 degradation through the proteasome pathway in both mouse and human HCC cell lines, as confirmed by the use of proteasome, lysosome and autophagy inhibitors. Network pharmacology analysis and molecular docking revealed that GSK-3β, a key regulator of PD-L1 degradation, is a target of emodin. Selective inhibitor-mediated suppression of GSK-3β largely reversed the regulatory effect of emodin on PD-L1. In contrast, overexpression of GSK-3β with a plasmid decreased PD-L1 protein levels and augmented emodin’s effect on PD-L1. Additionally, RNA-sequencing revealed the role of emodin in improving the immune responses in the tumor microenvironment. Finally, we observed a synergistic effect when the H_22_ cell subcutaneous tumor model was treated with emodin and anti-PD-L1 antibody.

**Conclusion:**

Emodin exerts anti-tumor effects by promoting GSK-3β-mediated PD-L1 proteasomal degradation and enhancing the anti-tumor effects of CD8^+^ T cells, indicating that emodin may be a promising therapeutic option for HCC.

**Graphical Abstract:**

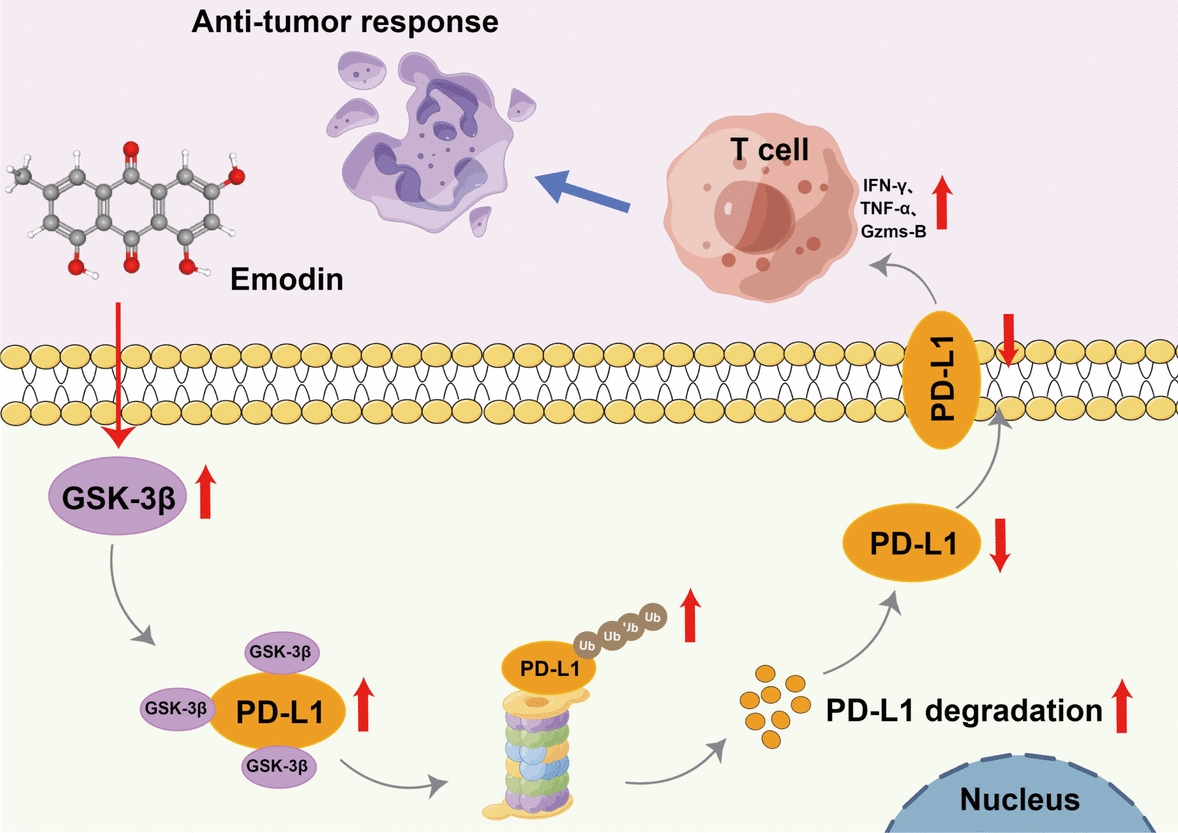

**Supplementary Information:**

The online version contains supplementary material available at 10.1186/s13020-025-01146-6.

## Introduction

Hepatocellular carcinoma (HCC) is the predominant type of liver cancer and accounts for 80–90% of all cases[1]. Globally, approximately 800,000 people died from HCC in 2020, and this number is projected to reach 1.2 million in 2040[2]. The main risk factors for HCC are chronic hepatitis B and C infections, alcohol use, and non-alcoholic steatohepatitis[3]. Clinically, liver resection, transplantation, and local ablation can cure most early-stage, nonmetastatic HCC cases[3]. Nevertheless, the majority of HCC patients are diagnosed in advanced stages, where curative treatment options are restricted and the prognosis is often unfavorable[4]. Therefore, effective treatment strategies for HCC are urgently needed.

Immune checkpoint inhibitors (ICIs), such as Atezolizumab (one of the anti-PD-L1 drugs), are now the preferred treatment for patients with unresectable HCC, which show good efficiency than chemotherapy or targeted therapies[5]. PD-L1, a prominent immune checkpoint, is predominantly found on the surface of tumor cells. PD-L1 interacts with PD-1 on tumor-infiltrating CD8^+^ T cells, triggering coinhibitory signals that lead to T cell exhaustion and immune escape[6]. PD-L1-targeting therapies have yielded significant benefits for patients with different solid tumors, including HCC[7]. However, only a small population of patients with unresectable HCC show promising clinical responses to anti-PD-L1 treatment, and many patients do not benefit from anti-PD-L1 treatment or discontinue treatment due to very serious side effects[8]. Therefore, new treatment approaches that can increase the efficacy and reduce the toxicity of anti-PD-L1 therapies in HCC patients are urgently needed.

Emodin (6-methyl-1,3,8-trihydroxyanthraquinone) is a natural anthraquinone compound derived from Chinese medicinal plants such as *Polygonum cuspidatum, Rheum palmatum L,* and *Polygonum multiflorum*[9]. A series of studies revealed that emodin can regulate the inflammatory response and has anti-inflammatory, antibacterial, and immune-stimulating effects[10]. Besides, emodin also has different degrees of anti-tumor activity in liver cancer[11], breast cancer[12], and lung cancer[13], which mainly results from its ability to directly hinder tumor cell growth and increase tumor cell death. Previous investigations revealed that emodin can diminish TNF-α–mediated PD-L1 stabilization or enhance the tumor-killing effects of CD8^+^ T cells in patients with breast cancer[12, 14]. These results strongly suggest that emodin has the potential to enhance anti-tumor effects by modulating PD-L1 expression. Nevertheless, whether emodin can regulate PD-L1 expression and its influence on anti-tumor immunity in HCC remains unclear.

In this study, we first determined that emodin can reduce PD-L1 expression in H_22_ cells or H_22_ cell subcutaneous tumor model. We found that emodin treatment enhanced anti-tumor effects by increasing CD8^+^ T cells infiltration and promoting TNF-α, IFN-γ, and granzyme B (Gzms-B) secretion. Mechanistically, emodin accelerated PD-L1 degradation through the proteasome pathway in both mouse and human HCC cell lines by targeting GSK-3β. Additionally, RNA-sequencing revealed that emodin may improve the immune response in HCC tumor microenvironment. Finally, we observed a synergistic effect when the H_22_ cell subcutaneous tumor model was treated with emodin and an anti-PD-L1 antibody. Taken together, our data strongly indicate that emodin represents a promising avenue for HCC treatment.

## Materials and methods

### Cell culture

The H_22_ mouse HCC cell line, HCC-LM3 (LM3) and Huh7 human HCC cell lines were obtained from Type Culture Collection of the Chinese Academy of Sciences (Shanghai, China). H_22_ cells were cultured in RPMI 1640 medium, and LM3 and Huh7 cell lines were cultured in DMEM supplemented with 10% FBS at 37 °C and 5% CO_2_.

### Quantitative real-time PCR analysis

Total RNA was extracted from cells and tumor tissues using an RNA isolation kit (#ER501-01; TransGen). First-strand complementary DNA (cDNA) was synthesized using the gDNA Eraser (#RR047a; Takara). PD-L1 and GAPDH mRNA levels were measured using the SYBR® Premix Ex Taq™ II (Tli RNaseH Plus) kit (#RR820a; Takara). Table 1 listed the primer sequences used in this investigation.

**Table 1:** Special primers used in qRT-PCR

Genes	Primer Sequence (5’–3’)
Mouse-PD-L1	F: TGCGGACTACAAGCGAATCACG R: CTCAGCTTCTGGATAACCCTCG
Mouse-GAPDH	F: GGTTGTCTCCTGCGACTTCA R: TGGTCCAGGGTTTCTTACTCC
Human-PD-L1	F: TGCCGACTACAAGCGAATTACTG R: CTGCTTGTCCAGATGACTTCGG
Human-GAPDH	F: CAGGAGGCATTGCTGATGAT R: GAAGGCTGGGGCTCATTT

### Western blotting

Western blot was performed as previously reported[15]. Equal amounts of protein lysates were divided using SDS-PAGE and transferred to PVDF membranes. After blocking for 1 h with 5% BSA, membranes were incubated with primary and secondary antibodies. Immunoreactivity was detected using an ECL kit (CST).

### TUNEL staining

Tumor cell apoptosis was detected using TUNEL staining as previously described[16]. Following dewaxing, antigen retrieval, and permeabilization, tumor sections were incubated in TUNEL reaction solution for 2 h at 37 °C. DAPI staining was then used to visualize nuclei. Images were captured utilizing a fluorescence microscope.

### Immunohistochemistry (IHC) and immunofluorescence (IF)

IHC and IF analyses were performed according to established protocols[17]. Primary antibodies included anti-Ki67 (Bioss, bsm-52455R), anti-CD8a (Abcam, ab217344), anti-GSK-3β (Proteintech, 82061), and anti-PD-L1 (Proteintech, 66248). Secondary antibodies were CoraLite488 goat-anti-rabbit IgG (Proteintech, SA00013-2) or CoraLite594 goat-anti-mouse IgG (Proteintech, SA00013-3).

### Flow cytometry

The H_22_-bearing subcutaneous tumor tissues were rapidly excised, mechanically dissociated, digested, and filtered to obtain single-cell suspensions. After excluding dead cells with DAPI, the enriched cells were stained with CD3-APC, CD4-PE, CD45-FITC, CD8-PE-Cy7, NK-1.1-PE, and F4/80-Pacific Blue™ for 20 min at room temperature. The cells were harvested and stimulated for 4 h with 2.5μL cell activation cocktail (composed of PMA, ionomycin, and Brefeldin A) (#423303; BioLegend) to assess T-cell effector function. After fixation, permeabilization, and intracellular labeling, the percentages of IFN-γ-APC, TNF-α-PE, or Granzyme B (Gzms-B)-FITC in CD8^+^ T cells were determined by flow cytometry. Data were analyzed using Beckman’s CytExpert software.

### Network pharmacology analysis

Network pharmacology was conducted as previously reported[18]. PharmMapper server, Similarity ensemble approach, and Swiss Target Prediction database were utilized to identify the potential bioactive ingredients of emodin. Human Gene Database GeneCards was used to identify genes related to HCC. Candidate targets of emodin against HCC were obtained by overlapping predicted targets with HCC-related genes. The STRING database was used to construct the PPI network. Network analysis was displayed using Cytoscape software (3.7.2), while Metascape was employed for conducting GO enrichment and KEGG pathway analyses.

### Molecular docking and molecular dynamics (MD) simulation

The GSK-3β crystal structure (PDB ID: 7B6F) and the three-dimensional structure of emodin (PubChem CID: 3220) were prepared for docking with AutoDock Vina 1.2.3. Small molecule and protein were described with GAFF2 and ff14SB force fields, respectively. The simulation included energy minimization, heating, equilibration, and a 100 ns production run. Binding free energies were calculated with the MM/GBSA method from 90 to 100 ns MD traces.

### Biotin-streptavidin pull-down assay

Emodin-binding proteins were identified using a biotin-streptavidin pull-down assay as previously described [19]. Briefly, proteins were extracted from lysed H_22_ and LM3 cells. Biotinylated emodin (#R-CW-DHB01, Ruixibio) or biotin control was then separately added to the cell lysates and incubated overnight at 4 °C. Subsequently, streptavidin magnetic beads (#HY-K0208, MCE) were added and the mixtures were incubated for 1 h at room temperature to allow binding of biotinylated emodin-protein complexes. After extensive washing to remove non-specific binding proteins, the pulled-down proteins were detected by western blot analysis.

### Inhibition or overexpression of GSK-3β

Cells were treated with 20 μM GSK-3β inhibitor TDZD-8 (Selleck) for 24 h for GSK-3β inhibition. For overexpression, cells were transfected with 2.5 μg of plasmid mixed with Opti-MEM medium and Lipofectamine 8000 reagent, followed by incubation for 24 h. The overexpression plasmids, Control PCMV and Flag-GSK-36, were provided by OBiO Technology (Shanghai Co., Ltd.).

### Animal models and treatment

Female BALB/c mice (6–8-week-old, 18 ± 2 g each) were randomly allocated into two groups (n = 8). After receiving a subcutaneous injection of 5 × 10^5^ H_22_ cells, the mice received an internal administration of emodin (50 mg/kg, daily) or saline (0.3% dimethyl sulfoxide [DMSO] and 5% Tween-80). The tumor size was assessed on day 5 after injection and calculated as follows: 0.5 × length × width^2^. The tumor weights were recorded at the endpoints.

### Statistical analysis

Statistical analyses were performed with GraphPad Prism 8. Statistical significance was determined by one-way ANOVA or unpaired Student's *t*-test. The criteria for statistical significance were set at *P* < 0.05 and *P* < 0.005.

## Results

### Emodin inhibits PD-L1 expression in HCC cells in vivo and in vitro

**Fig. 1:**
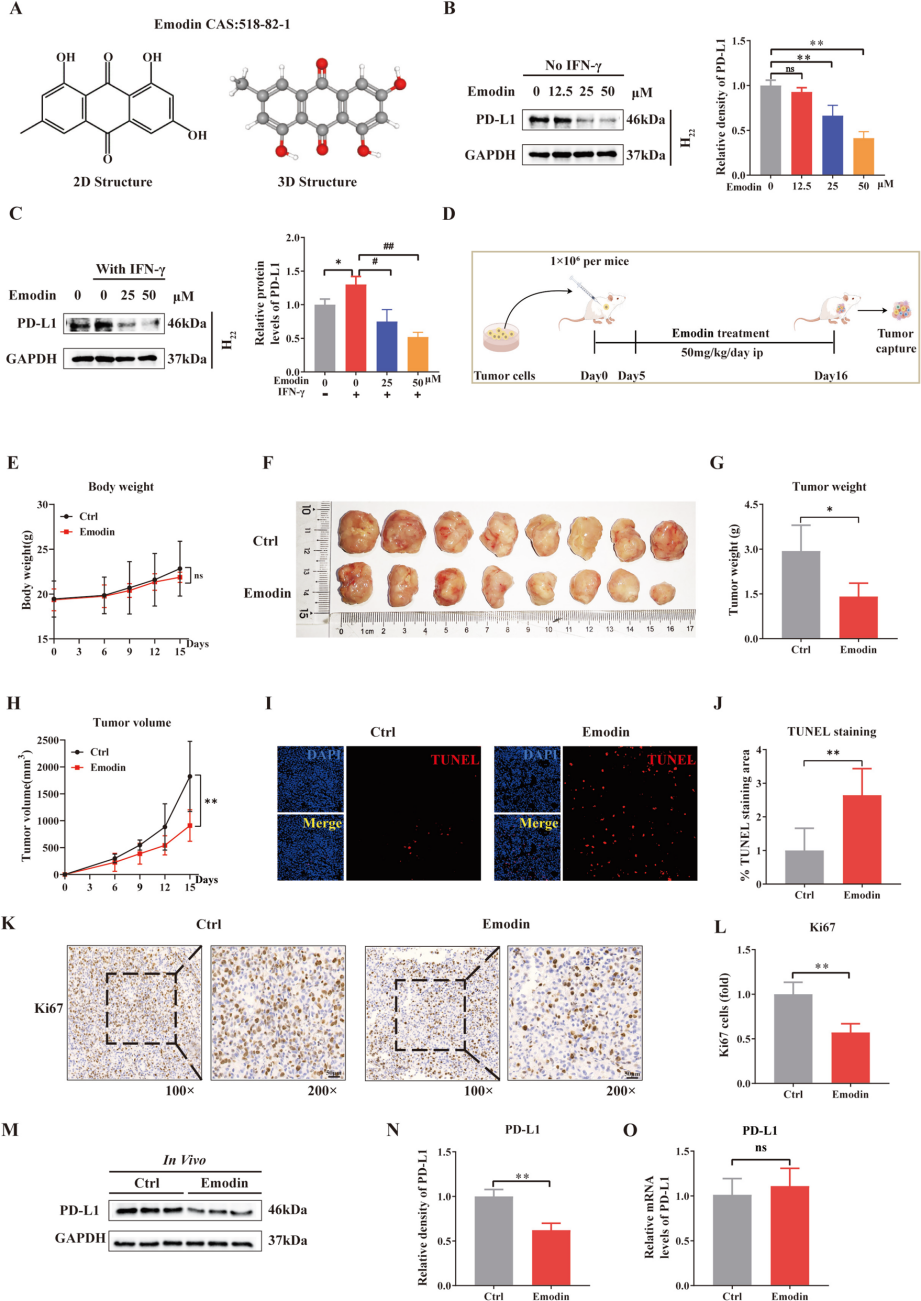
Emodin inhibits PD-L1 expression in HCC cells in vivo and in vitro. **A** 2D and 3D chemical structures of emodin. **B** H_22_ cells were treated with emodin at different concentrations (12.5, 25, 50 μM) for 24 h and PD-L1 expression was analyzed using western blotting. **C** H_22_ cells were treated with different concentrations (25, 50 μM) of emodin for 24 h with IFN-γ stimulation, and PD-L1 expression was analyzed using western blotting. **D** Schematic representation of the animal models and treatment plan for emodin. **E** Body weight. **F** Images of typical tumors. **G** Weight of the tumors. **H** Tumor size was recorded at the indicated times. **I**, **J** TUNEL staining of the tumor tissues and statistical results. (Scale bar: 50 μm, × 200 magnification). **K**, **L** Ki67 staining of tumor tissues and statistical results. (Scale bar: 50 μm, × 200 magnification). **M**, **N** Western blot analysis of the protein levels of PD-L1 in tumor tissues and the statistical results. **O** RT-qPCR was used to measure the mRNA expression of PD-L1 in tumor tissues. All data are shown as mean ± SD and Student’s t test was used for comparison. *P < 0.05, **P < 0.01 vs. controls. ^#^*P* < 0.05, ^##^*P* < 0.01 vs IFN-γ group

The 2D and 3D chemical structures of emodin are displayed in Fig. 1A. First, we asked whether emodin can reduce PD-L1 expression in vitro. H_22_ HCC cells were exposed to emodin at concentrations of 0, 12.5, 25 or 50 μM, and emodin decreased PD-L1 expression at concentrations of 25 and 50 μM, but not at 12.5 μM. (Fig. 1B). IFN-γ, which is secreted by infiltrated cytotoxic T lymphocytes (CTLs), is a strong inducer of PD-L1 in the tumor microenvironment[20]. Therefore, we focused on whether emodin attenuated IFN-γ-induced PD-L1 expression in H_22_ cells in vitro. Similarly, emodin reduced PD-L1 levels in an IFN-γ-induced manner (Fig. 1C).

To detect the anti-HCC and anti-PD-L1 effects of emodin in vivo, we used an H_22_ subcutaneous-bearing BALB/c mouse model and treated the mice with emodin (50 mg/kg/day) for 10 consecutive days, as illustrated in Fig. 1D. No difference in body weight between the control and emodin groups was observed (Fig. 1E). However, emodin reduced tumor size and tumor weight in H_22_ subcutaneous tumor-bearing mice compared with those in the control group (Fig. 1F, G). Compared with the control group, the emodin-treated group had significantly smaller tumor volumes (Fig. 1H). TUNEL staining revealed that emodin markedly increased the percentage of apoptotic cells in the subcutaneous tumor samples (Fig. 1I, J). Moreover, Ki67 staining revealed that emodin suppressed the proliferation of subcutaneous tumor samples (Fig. 1K, L). Next, western blot analysis was performed to detect PD-L1 expression in subcutaneous tumor tissues, and the results revealed that emodin significantly reduced PD-L1 expression in vivo (Fig. 1M, N). Intriguingly, we detected no change in the mRNA levels of PD-L1 (Fig. 1O).

In summary, emodin reduced PD-L1 protein levels in vivo and in vitro, and inhibited tumor growth in an H_22_ subcutaneous tumor-bearing BALB/c mouse model.

### Emodin treatment enhances CD8^+^T cell infiltration and improves anti-tumor immunity in HCC

**Fig. 2:**
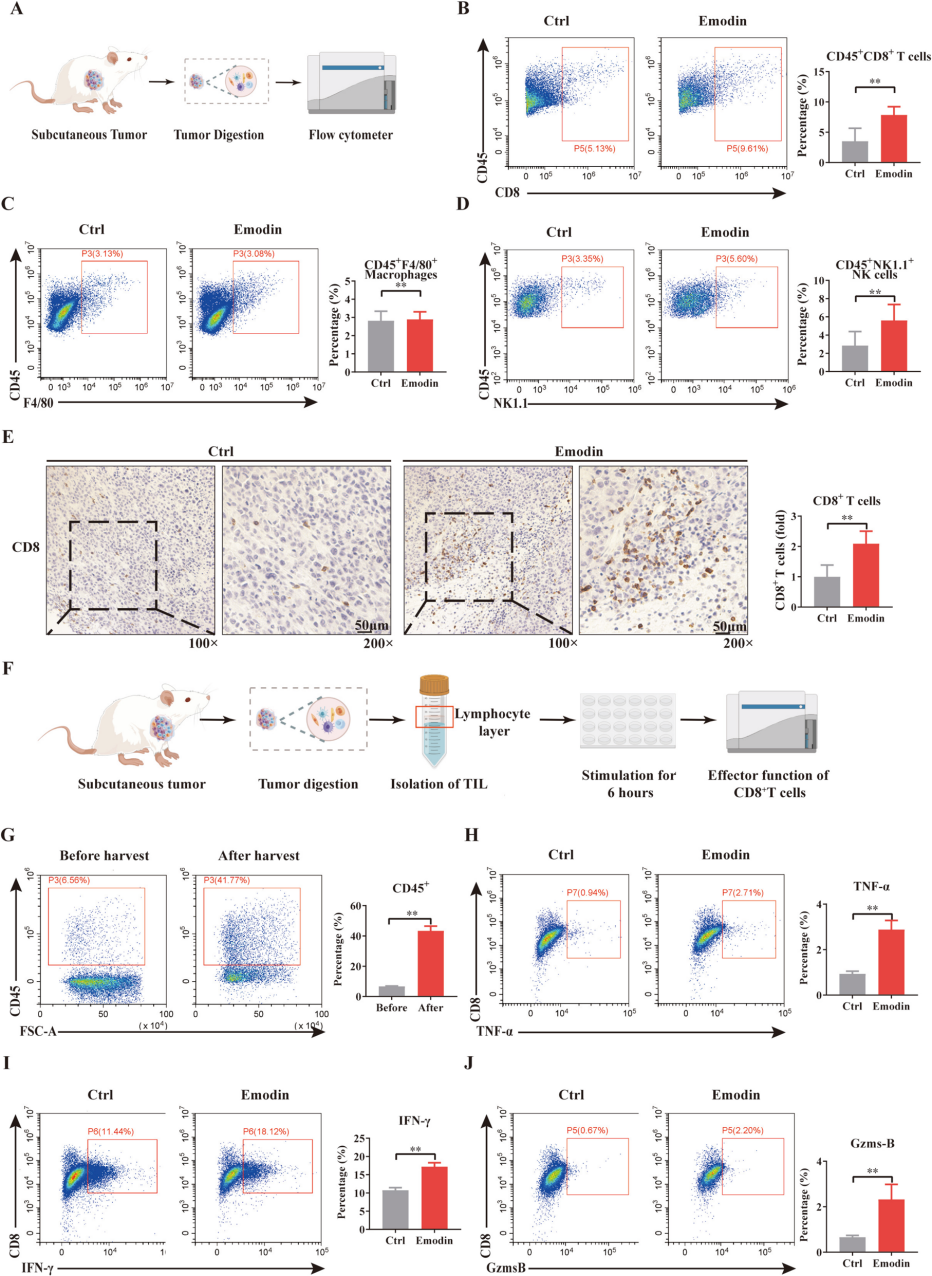
Emodin treatment enhances CD8^+^T cell infiltration and improves anti-tumor immunity in HCC. **A** Schematic illustration of flow cytometry analysis of subcutaneous tumors. The proportions of **B** CD8^+^T, **C** macrophages, and **D** NK cells after tumor digestion. **E** Immunohistochemical staining of CD8. **F** A schematic for evaluating the effector functions of cytotoxic CD8^+^T cells in subcutaneous tumors. **G** Harvested immune cells from heterogeneously digested tumor samples. **H**–**J** Flow cytometry was used to evaluate the expression of TNF-α (**H**), IFN-γ (**I**), and Gzms-B (**J**) in tumor-infiltrating CD8^+^T cells. Data were from three independent experiments (mean ± SD, **P < 0.01 vs. controls)

Logically, PD-L1 inhibition in the tumor environment always implies a good outcome for anti-tumor immunity. Thus, we harvested tumor samples and obtained cell suspensions for flow cytometry analysis, as illustrated in Fig. 2A. Flow cytometry detection was performed on anti-tumor immune cells, including macrophages, CD8^+^ T cells, and natural killer (NK) cells. We found that there was no difference in the percentages of macrophages between the control and emodin groups (Fig. 2C), but there were significant increases in the percentages of CD8^+^ T cells and NK cells (Fig. 2B and D). Immunohistochemical assays also revealed that emodin increased the infiltration of CD8^+^T cells (Fig. 2E).

CD8^+^ cytotoxic T cells are the most powerful tumor-killing immune cell, they act by releasing cytotoxic molecules IFN-γ, TNF-α and Gzms-B. Thus, in the next experiment, intracellular staining of these effective molecules in CD8^+^ T cells was performed. Given the limited number of CD8^+^ T cells in tumors, we harvested immune cells from heterogeneously digested tumor samples using a mouse lymphocyte isolation solution (Fig. 2F). The percentage of CD45^+^ cells increased from 6.74 ± 0.20% to 43.39 ± 3.08%, which is sufficient for in vitro stimulation. After stimulation with a cell activation cocktail, emodin upregulated the expression of TNF-α (Fig. 2H), IFN-γ (Fig. 2I) and Gzms-B (Fig. 2J).

Together, these results indicate that emodin enhances anti-tumor immune response to HCC by promoting CD8^+^ T cells infiltration and increasing the secretion of effector molecules.

### Emodin accelerates PD-L1 degradation in the proteasome

**Fig. 3:**
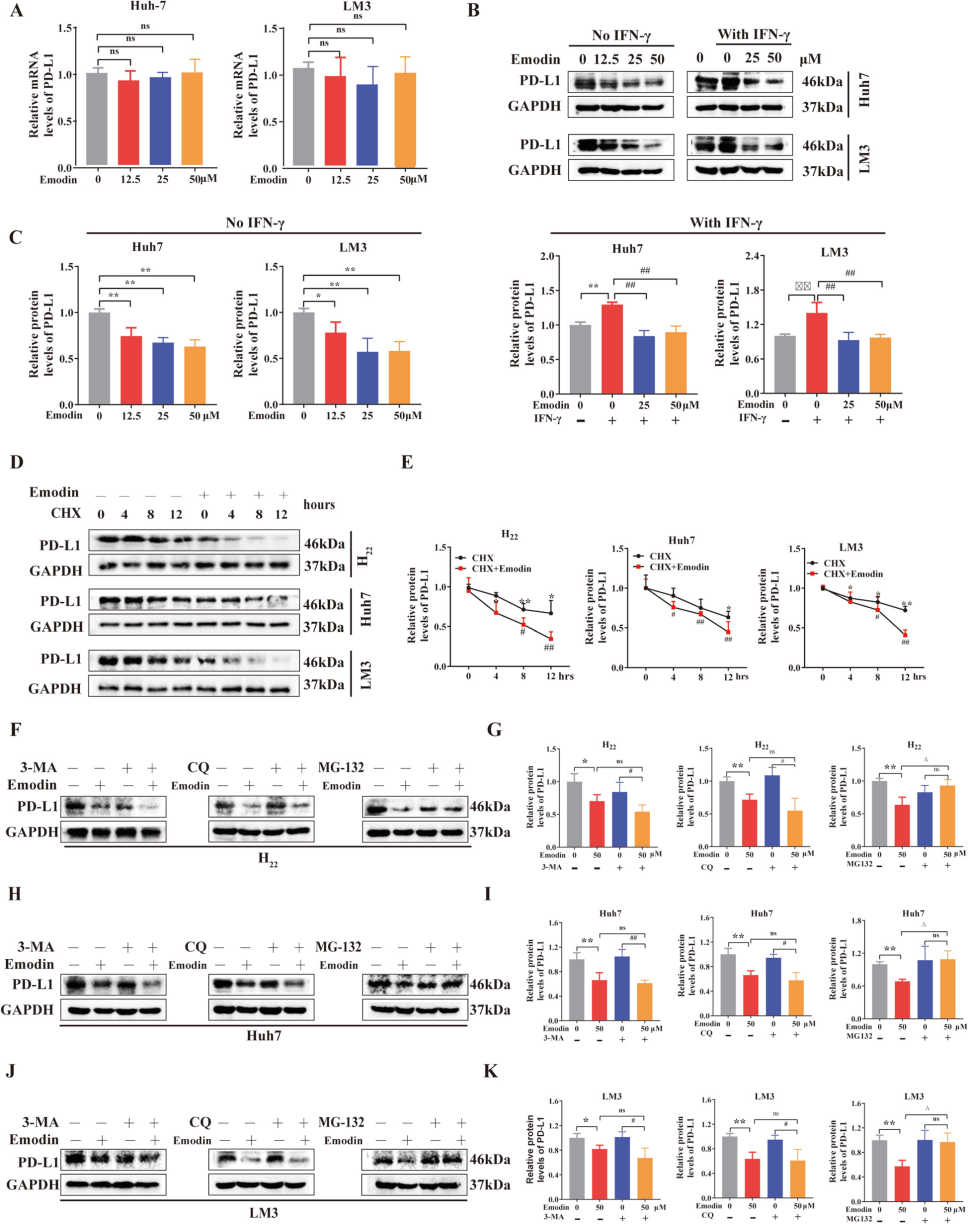
Emodin accelerates PD-L1 degradation in proteasomes. **A** PD-L1 mRNA and **B**, **C** protein levels in human HCC Huh7, and LM3 cells cultured for 24 h with 12.5, 25, or 50 µM emodin with or without IFN-γ exposure. **D**, **E** PD-L1 protein half-life was assessed using the CHX chase assay. The cells were treated with emodin for 24 h before being exposed to CHX at the specified times. **F**, **G** H_22_, **H**, **I** Huh7, and **J**, **K** LM3 cells were first exposed to emodin (50 µM) and then incubated for 24 h with 3-MA (5 mM), CQ (20 µM), or MG132 (10 µM). Then, the PD-L1 protein level was measured. Data are the mean ± SD of three independent experiments. *P < 0.05, **P < 0.01 vs. controls. ^△^*P* < 0.05 vs. emodin group. ^#^*P* < 0.05, ^##^*P* < 0.01 vs. 3-MA, CQ, or MG-132 group

To validate the findings of mouse and human studies, we first assessed whether emodin could decrease PD-L1 expression in Huh7 and LM3 cells (two human HCC cell lines). qRT-PCR was used to examine changes in PD-L1 mRNA expression after emodin treatment (Fig. 3A). To our surprise, emodin treatment had no effect on PD-L1 mRNA levels. Next, Huh7 and LM3 cells were incubated with or without IFN-γ and exposed to emodin at concentrations of 12.5, 25 and 50 μM. Like in mouse H_22_ cells, emodin reduced PD-L1 expression in a concentration-dependent manner (Fig. 3B, C). In summary, the findings above indicate that emodin may decrease PD-L1 expression through posttranscriptional regulation. To test this hypothesis, cycloheximide (CHX), a well-known protein synthesis inhibitor, was used to induce PD-L1 degradation. Emodin treatment led to a substantial reduction in the half-life of the PD-L1 protein in mouse H_22_ cells or human Huh7 and LM3 cells, indicating that emodin significantly enhanced the degradation of PD-L1 (Fig. 3D and E). PD-L1 is typically degraded in proteasomes or lysosomes, a process regulated by various signaling pathways[21]. Here, we employed chloroquine (CQ) as a lysosomal inhibitor, 3-MA as an autophagy inhibitor, and MG132 as a proteasome inhibitor to elucidate the detail mechanism by which emodin induces PD-L1 degradation. Interestingly, MG132 (but not CQ and 3-MA) suppressed emodin-induced PD-L1 downregulation in the H_22_, Huh7 and LM3 cell lines (Fig. 3F–K). The administration of emodin significantly promoted the polyubiquitination of PD-L1, as shown in Supplementary Fig. 1. Overall, these results demonstrated that emodin promotes PD-L1 degradation through a ubiquitin-mediated mechanism.

### Identification of potential therapeutic targets of emodin

**Fig. 4:**
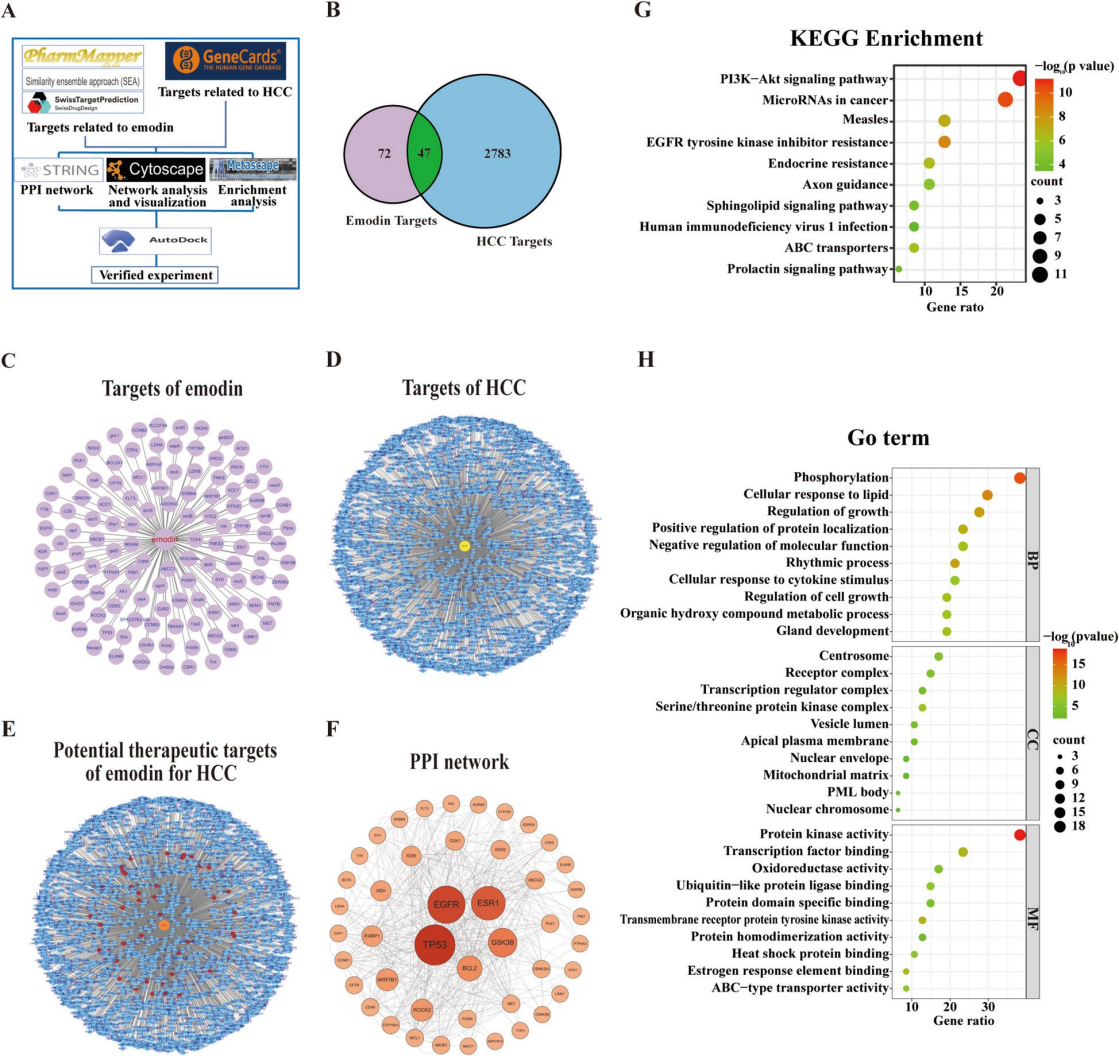
Network pharmacology analysis of emodin in HCC. **A** The network pharmacology analysis is depicted in a flowchart, illustrating the process. **B** Venn diagram of the predicted target genes of emodin in HCC. **C**, **D** Identification of the molecular targets of emodin and HCC. **E** Potential therapeutic targets of emodin specific to HCC. **F** The PPI network of potential therapeutic targets of emodin in HCC. **G**, **H** KEGG and GO enrichment analyses, respectively

Network pharmacology was used to predict emodin’s potential targets for treating HCC (Fig. 4A). Databases were used to screen and integrate ‘drug targets’ and ‘disease targets’. Data integration using PharmMapper, the Similarity Ensemble Approach, and SwissTargetPrediction identified 72 targets associated with emodin (Fig. 4B). Following the removal of duplicates, 2783 HCC-related targets were obtained from the GeneCards database (Fig. 4B). Among these, 47 shared targets were identified as potential targets of emodin for treating HCC. (Fig. 4B and E). The 31 possible therapeutic targets mentioned earlier were further examined using the STRING database. The information was then imported into Cytoscape v3.7.1 software to create an interaction network and five core targets, namely, GSK3β, TP53, EGFR, ESR1, and BCL2, were identified via topology analysis (Fig. 4F). GO and KEGG enrichment analyses revealed the ubiquitin–like protein ligase binding and PI3K/Akt signaling pathways (Fig. 4G, H). Hyperactivation of the PI3K/AKT pathway leads to GSK3β inactivation, which in turn prevents the degradation of PD-L1 by GSK3β. This leads to increased expression of PD-L1 on malignant liver cells, facilitating tumor immune evasion via the PD-L1/PD-1 axis and, ultimately, HCC development[22]. Therefore, we hypothesized that emodin might exert its effects by regulating GSK3β-mediated PD-L1 degradation.

### Simulation analysis of the interaction between emodin and GSK-3β

**Fig. 5:**
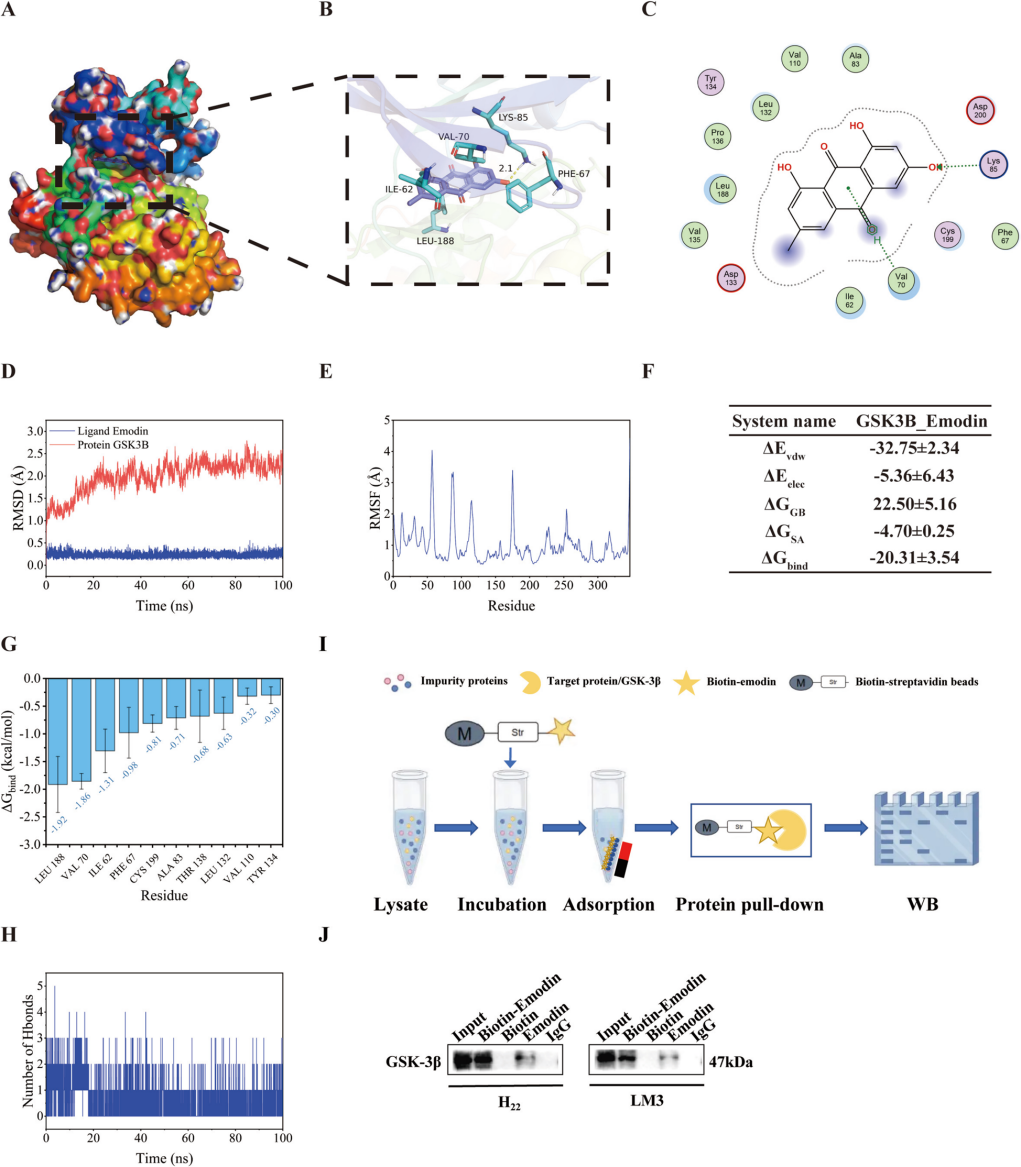
Interactions between emodin and GSK-3β. **A**–**C** Molecular models of the binding of the selected compound emodin to GSK-3β. **A** Overall view of small molecule protein binding, **B** Partial view, with a blue stick symbolizing a small molecule and a light blue cartoon symbolizing the amino acid binding sites, the yellow dashed line symbolizes hydrogen bonding. **C** 2D interaction diagram. **D**–**H** Molecular dynamics simulations. **D** The RMSD plot of GSK-3β-emodin. **E** The RMSF of GSK-3β-emodin. **F** The binding affinity and related energetics were estimated using MM/GBSA (kcal/mol). **G** The top 10 amino acids involved in the interaction between emodin and GSK-3β. **H** The number of hydrogen bonds between emodin and GSK-3β. **I** Schematic diagram of the biotin-streptavidin pull-down assay. **J** Pull-down experiments were performed to detect the binding of emodin to GSK-3β

Molecular docking is an effective method for exploring small molecule-protein interactions. We utilized Vina 1.2.3 to dock emodin with the GSK-3β protein. As shown, emodin bound within the internal active site of GSK-3β (Fig. 5A). Comprehensive analysis of the interactions revealed that the binding site is composed of LEU-132, PRO-136, LEU-188, VAL-135, ILE-62, VAL-70, ASP-133, CYS-190, LYS-199, LYS-85, and ASP-200. Emodin engages in hydrophobic interactions with LEU-132, PRO-136, LEU-188, VAL-135, ILE-62, and VAL-70 and forms hydrogen bonds with LYS-85 (Fig. 5B, C). The binding affinity score of -9.195 kcal/mol suggests a strong potential for interaction.

On the basis of the molecular docking results, emodin and GSK-3β were selected for the MD simulations. The RMSD of the GSK-3β-emodin complex remained between 0 and 3 Å over a 200 ns period, indicating stability (Fig. 5D). Per-residue RMSF analysis (Fig. 5E) revealed minimal fluctuations, suggesting that the complex’s binding site was stable. MM-GBSA calculations yielded a binding energy of -20.31 ± 3.54 kcal/mol (Fig. 5F). The top 10 amino acids contributing to small molecule and protein binding, reflected strong affinity (Fig. 5G). Although the number of hydrogen bonds between the ligand and protein ranged from 1 to 5 (Fig. 5H), their contribution to binding stability was relatively low. These results confirmed the stable conformation of the complex throughout the simulation. Furthermore, we performed a biotin-streptavidin pull-down assay to evaluate the binding of emodin to GSK-3β, as illustrated in Fig. 5I. GSK-3β was pulled down by biotin-labeled emodin, suggesting a direct interaction between emodin and GSK-3β (Fig. 5J).

### Emodin directly targets GSK-3β to accelerate PD-L1 degradation

**Fig. 6:**
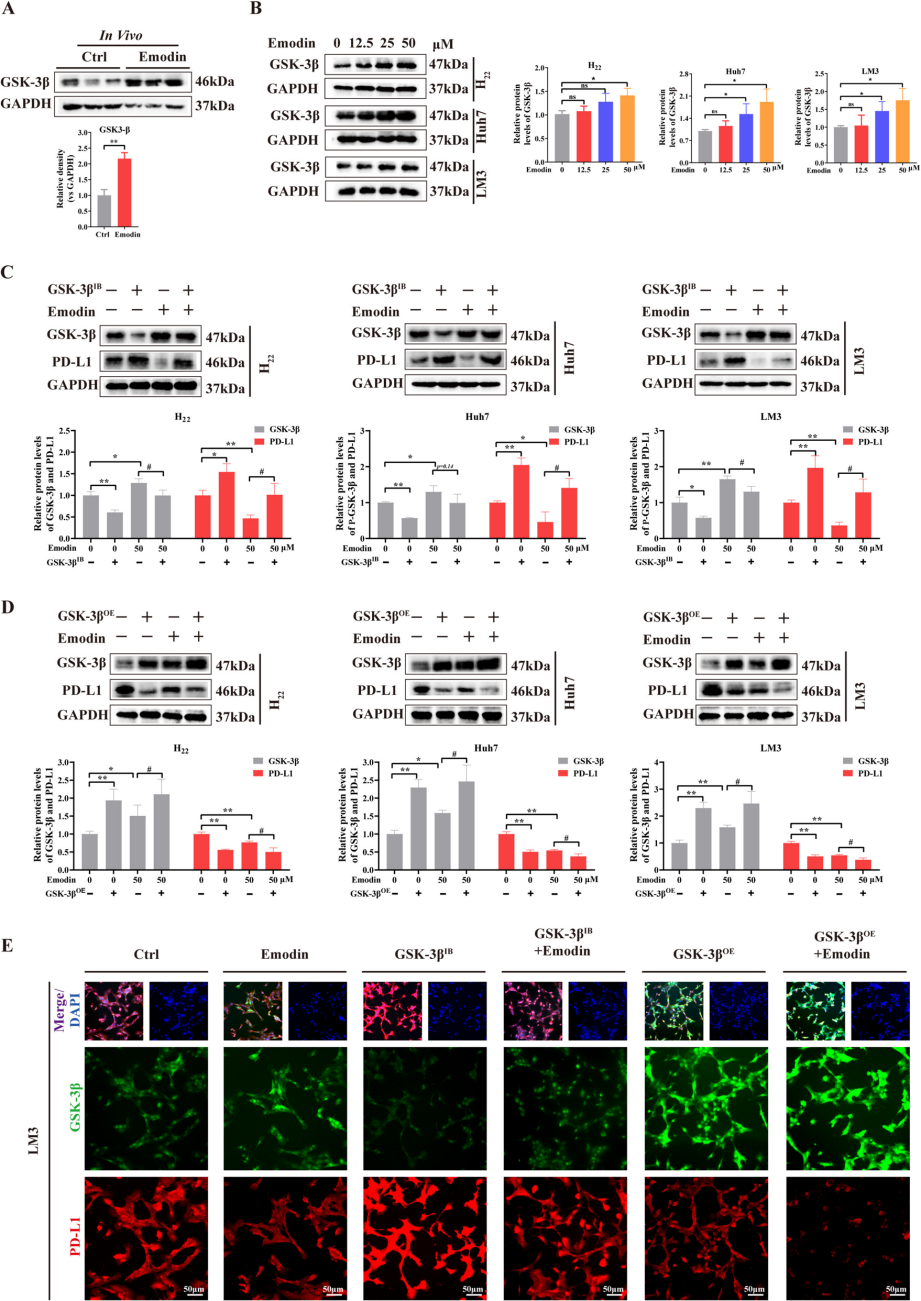
Emodin directly targets GSK-3β to accelerate PD-L1 degradation. **A**, **B** GSK-3β protein expression compared with that of GAPDH in tumor tissues, H_22_, Huh7, and LM3 cells after emodin treatment. **C** Exposure of H_22_, Huh7, and LM3 cells with GSK-3β inhibition (GSK-3β^IB^) to emodin. Western blot analysis was conducted to measure the expression levels of GSK-3β and PD-L1. **D** Overexpressing GSK-3β (GSK-3β^OE^) enhanced the inhibitory effects of emodin on PD-L1 protein expression in H_22_, Huh7, and LM3 cells. **E** Fluorescence images of LM3 cells that were stained with anti-GSK-3β (green), anti-PD-L1 (red), and DAPI (blue) (200 ×). The data are from three independent experiments (mean ± SD, *P < 0.05, **P < 0.01 vs. controls. ^#^*P* < 0.05 vs. emodin group)

Moreover, western blotting demonstrated that the emodin treatment group presented considerably higher GSK-3β expression than the control group in vivo and in vitro (Fig. 6A, B). The influence of GSK-3β on PD-L1 expression was investigated via the use of TDZD-8, a selective GSK-3β inhibitor. The findings revealed that inhibiting GSK-3β (GSK-3β^IB^) increased PD-L1 protein levels as expected and ablated the effect of emodin on PD-L1(Fig. 6C). In contrast, overexpressing GSK-3β (GSK-3β^OE^) using plasmid transfection decreased the PD-L1 protein level. Concomitant treatment with emodin and the GSK-3β overexpression plasmid synergistically suppressed PD-L1 expression in HCC cells (Fig. 6D). Similar results were obtained via immunofluorescence staining of GSK-3β and PD-L1 after emodin treatment (Fig. 6E). These results demonstrated that emodin accelerated PD-L1 degradation by targeting GSK-3β.

### RNA-sequencing results and combination treatment with emodin and anti-PD-L1 antibody in vivo

**Fig. 7:**
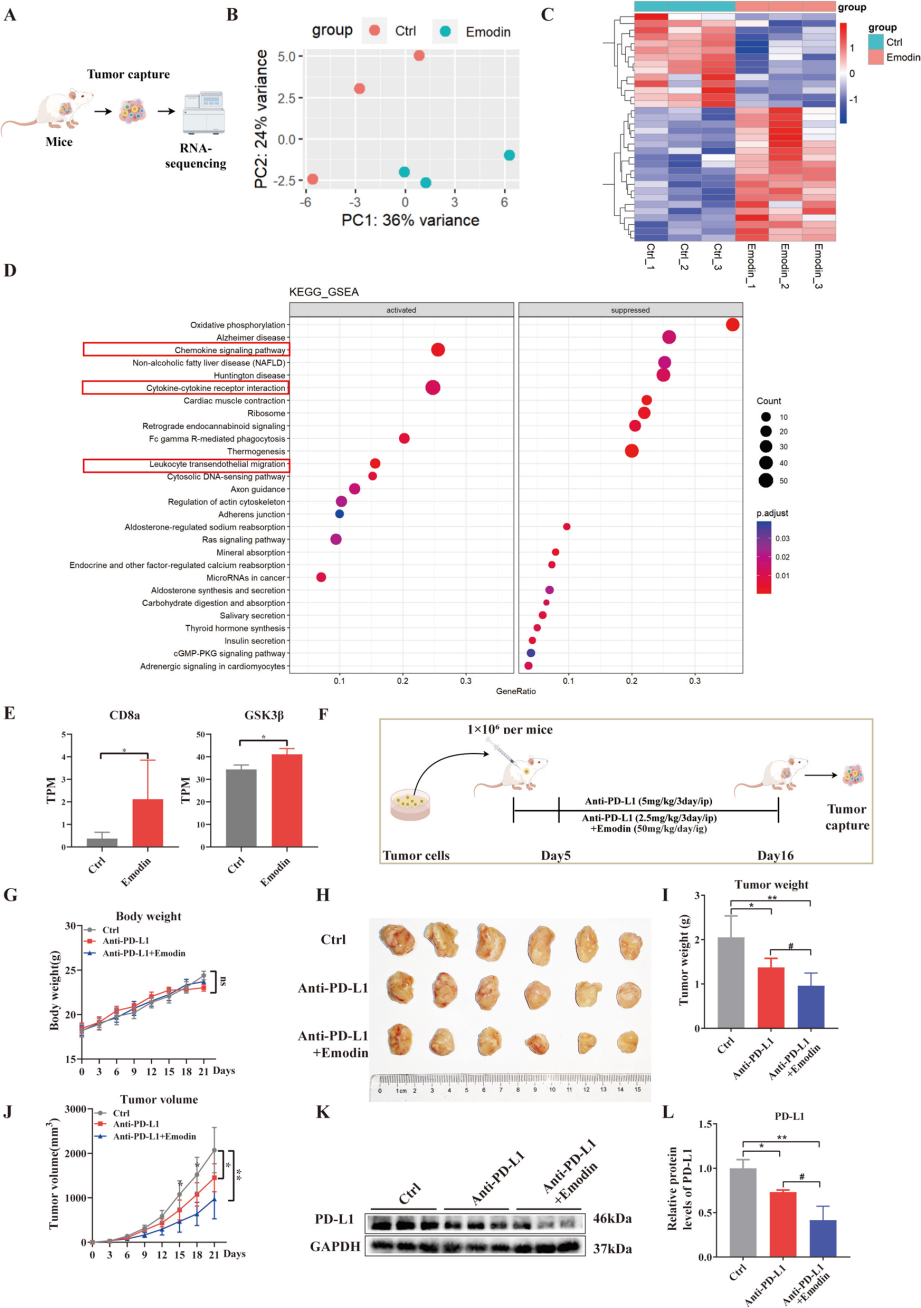
RNA-sequencing results and combination treatment with emodin and anti-PD-L1 antibody in vivo. **A** Schematic of the RNA-sequencing analysis. **B** Principal component analysis. **C** Heatmap of 34 identified differential genes. **D** Gene Set Enrichment Analysis. **E** Transcript per million values of CD8a and GSK-3β. **F** Schematic representation of the animal models and treatment plans of emodin and anti-PD-L1. **G** Body weight. **H** Images of typical tumors. **I** Weight of the tumors. **J** Tumor size was recorded at specified times. **K**, **L** Western blot analysis of the protein levels of PD-L1 in tumor tissues and statistical results. The data are from three independent experiments (mean ± SD, *P < 0.05, **P < 0.01 vs. controls. ^#^*P* < 0.05 vs. Anti-PD-L1 group)

To further verify the results mentioned above, subcutaneous tumor samples were sent for bulk RNA-sequencing analysis (Fig. 7A). The results from RNA-sequencing revealed an increase in the expression of 20 genes and a decrease in the expression of 14 genes in the group treated with emodin (Fig. 7B, C). CD8a and GSK3β mRNAs were upregulated after emodin treatment compared with the transcript per million (TPM) value (Fig. 7E). Next, the differentially expressed genes were subjected to Gene Set Enrichment Analysis (GSEA). The analysis revealed enrichment in the chemokine signaling pathway, cytokine-cytokine receptor interaction pathway and leukocyte transendothelial migration pathway (Fig. 7D).

Next, we investigated whether emodin and anti-PD-L1 therapy have synergistic effects on HCC. H_22_ subcutaneous tumor-bearing mice were treated with anti-PD-L1 (5 mg/kg every 3 days) or anti-PD-L1 (2.5 mg/kg every 3 days) combined with emodin (50 mg/kg/daily), as displayed in Fig. 7F. We observed no significant difference in body weight among the control, anti-PD-L1, and emodin plus anti-PD-L1 groups (Fig. 7G). Intriguingly, we found that emodin plus anti-PD-L1 therapy (2.5 mg/kg every 3 days) resulted in a smaller tumor size and tumor volume than anti-PD-L1 monotherapy (5 mg/kg every 3 days), suggesting that the combination use of emodin and anti-PD-L1 therapy has great potential to enhance anti-tumor efficacy and reduce the degree of dose related anti-PD-L1 toxicity (Fig. 7H–J). Additionally, we evaluated the combination of emodin and an anti-PD-1 antibody. Our results revealed that, compared with anti-PD-1 monotherapy, emodin plus anti-PD-1 therapy significantly reduced tumor size and weight, as shown in Supplementary Fig. 2. Finally, we evaluated PD-L1 expression by western blotting. The results revealed that emodin plus anti-PD-L1 (2.5 mg/kg every 3 days) had the lowest PD-L1 expression (Fig. 7K, L).

## Discussion

Emodin, a broad-spectrum anti-cancer agent, has exhibited therapeutic potential for several cancers, including breast cancer[23], non-small cell lung cancer[13], colon cancer[24] and gastric cancer[25]. Mechanistically, the majority of related studies have focused on its direct effects (eg. the inhibition of cancer cell growth and metastasis or the suppression of cancer cell proliferation) [26]. However, studies on the effects of emodin on anti-tumor immunity are still lacking. Our study revealed that emodin significantly decreased PD-L1 protein levels in vivo and in vitro by accelerating its degradation through the proteasome pathway. Consequently, emodin treatment enhanced the anti-tumor effects of emodin alone or in combination with anti-PD-L1 antibody.

PD-L1, which is also referred to as CD274 or B7-H1, is commonly expressed on the surface of various tumor cells. High PD-L1 levels in the tumor microenvironment indicate a poor prognosis for various tumors. PD-L1 is a recognized target for immune checkpoint blockade [27]. Multiple signaling pathways, including posttranscriptional and posttranslational regulation, regulate the expression of PD-L1 in tumor cells [21]. Existing evidence indicates that PD-L1 internalization and recycling are key reasons for anti-PD-L1 resistance [28]. Typically, PD-L1 is internalized after binding with an anti-PD-L1 antibody and tends to be degraded in proteasomes or lysosomes [28]. However, most internalized PD-L1 is recycled back to the cell membrane, which mediates resistance to ICIs [29]. Thus, targeting PD-L1 degradation can increase the efficacy of tumor immunotherapy [22, 30, 31]. In a previous study, Lim et al. discovered that emodin attenuated PD-L1 stabilization in breast cancer, but the detail mechanism was not explored [14]. In the present study, emodin decreased PD-L1 levels in both human and mouse HCC cell lines; this effect occurred mainly via the acceleration of PD-L1 degradation in the proteasome rather than via the regulation of mRNA levels.

GSK-3β, a member of the serine/threonine protein kinase family, was initially recognized as a controller of glycogen metabolism. An increasing number of studies have revealed the vital role of GSK3β in the development of HCC [32]. However, its functions in HCC remain controversial [33]. Several studies revealed that GSK-3β inhibition led to reduced viability, proliferation, metastasis and tumorigenic properties in HCC cells [34]. However, other studies indicate that GSK-3β functions as a tumor suppressor gene in HCC [33]. Our previous research indicated that increased GSK-3β expression was positively correlated with the cell death and metastasis of HCC cell lines, suggesting its role as a tumor suppressor in HCC [35, 36]. More importantly, GSK-3β can specifically bind to the C-terminal domain of nonglycosylated PD-L1 at the N219, N200 and N192 glycosylation sites, leading to phosphorylation-dependent degradation by the proteasome [37]. Man et al. reported that GSK3β accelerates PD-L1 degradation, resulting in lower levels of PD-L1 in HCC cells[22]. Thus, increased GSK-3β expression always implies increased PD-L1 degradation. In Qin’s study, emodin increased GSK-3β expression in a concentration-dependent manner [38]. Here, we identified GSK-3β as a target of emodin through network pharmacology analysis and molecular docking. Emodin promoted GSK-3β expression in both human and mouse HCC cell lines, and GSK-3β expression was inversely correlated with PD-L1 levels.

Tumor infiltrating cytotoxic T cells with surface CD8 expression, are the most potent tumor killing immune cells [39]. Clinically, CD8^+^ T cell-based immunotherapy has shown promising results in many patients [40]. Nevertheless, the activity of CD8^+^ T cells in the tumor microenvironment is always suppressed by different mechanisms. The binding of PD-L1 expressed by tumors to PD-1 on infiltrating CD8^+^ T cells triggers coinhibitory signaling, which is a key factor in CD8^+^ T cell exhaustion and tumor immune evasion [6]. Anti-PD-1/PD-L1 inhibitors have become effective and are rapidly becoming the primary treatment for various cancers, including HCC [5]. However, only a subset of patients with unresectable HCC show promising clinical responses to anti-PD-L1 treatment, and severe immune-related side effects restrict the clinical use of PD-L1 monoclonal antibodies [41]. Therefore, treatment strategies targeting the PD-L1/PD-1 axis, aimed at increasing anti-tumor immunity and reducing toxicity, still need to be developed. Here, we revealed that emodin treatment enhanced anti-tumor effects in an H_22_ subcutaneous tumor-bearing mice model by increasing CD8^+^ T cell infiltration and promoting TNF-α, IFN-γ, and Gzms-B secretion. In addition, we also found that emodin plus anti-PD-L1 (2.5 mg/kg every 3 days) led to a smaller tumor size and tumor volume than anti-PD-L1 (5 mg/kg every 3 days) monotherapy, suggesting that the combination of emodin and anti-PD-L1 has great potential to enhance anti-tumor efficacy and reduce dose-related anti-PD-L1 toxicity.

## Conclusions

**Fig. 8:**
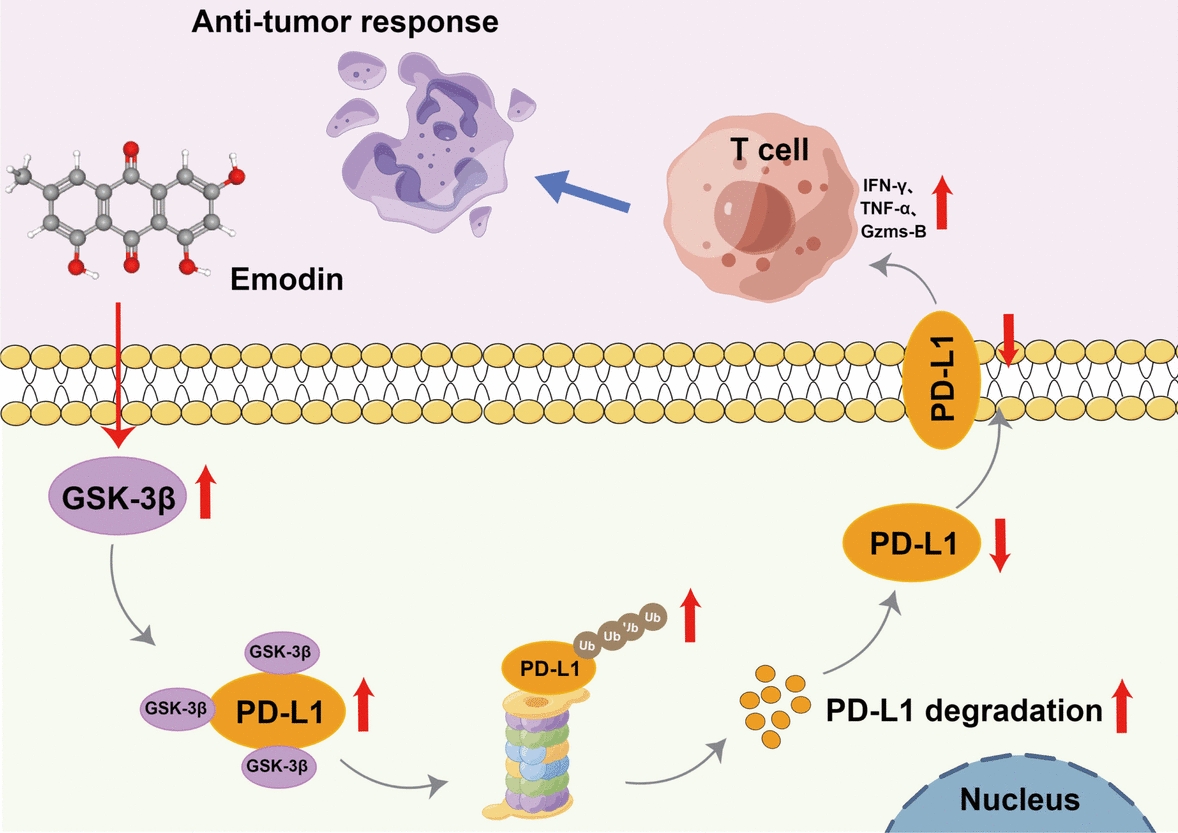
Schematic diagram of how emodin promotes PD-L1 degradation and enhances the anti-tumor effector function of CD8^+^ T cells. Emodin promotes ubiquitin-mediated PD-L1 degradation by inhibiting GSK-3β expression. Furthermore, emodin stimulates TNF-α, IFN-γ, and Gzms-B production in CD8^+^ T cells by enhancing their tumor-killing effect

The current research demonstrated that emodin can suppress PD-L1 expression and enhance anti-tumor immunity in HCC. Mechanistically, emodin accelerated PD-L1 degradation through the proteasome pathway in both mouse and human HCC cell lines by targeting GSK-3β, as illustrated in Fig. 8. Collectively, our results indicate that emodin may be a promising therapeutic option for HCC.

## Supplementary Information

Supplementary file 1.

## Data Availability

The research data used to support the fundings are available from the corresponding author upon request.

## Abbreviations

HCC: hepatocellular carcinoma
PD-L1: Programmed death-ligand 1
PD-1: programmed death protein-1
GSK-3β: glycogen synthase kinase-3 beta
ICIs: immune checkpoint inhibitors
Gzms-B: granzyme B
CTLs: infiltrated cytotoxic T lymphocytes
NK: natural killer
CHX: cycloheximide
CQ: chloroquine
TPM: transcript per million
GSEA: Gene Set Enrichment Analysis
MD: molecular dynamics
IHC: Immunohistochemistry
IF: immunofluorescence

## References

[1] Kim DY. Changing etiology and epidemiology of hepatocellular carcinoma: Asia and worldwide. J Liver Cancer. 2024;24(1):62–70. 38523466 10.17998/jlc.2024.03.13 PMC10990659

[2] Rumgay H, Arnold M, Ferlay J, Lesi O, Cabasag CJ, Vignat J, et al. Global burden of primary liver cancer in 2020 and predictions to 2040. J Hepatol. 2022;77(6):1598–606. 36208844 10.1016/j.jhep.2022.08.021 PMC9670241

[3] Llovet JM, Kelley RK, Villanueva A, Singal AG, Pikarsky E, Roayaie S, et al. Author correction: hepatocellular carcinoma. Nat Rev Dis Primers. 2024;10(1):10. 38346997 10.1038/s41572-024-00500-6

[4] Yang JD, Hainaut P, Gores GJ, Amadou A, Plymoth A, Roberts LR. A global view of hepatocellular carcinoma: trends, risk, prevention and management. Nat Rev Gastroenterol Hepatol. 2019;16(10):589–604. 31439937 10.1038/s41575-019-0186-y PMC6813818

[5] Finn RS, Qin S, Ikeda M, Galle PR, Ducreux M, Kim TY, et al. Atezolizumab plus bevacizumab in unresectable hepatocellular carcinoma. N Engl J Med. 2020;382(20):1894–905. 32402160 10.1056/NEJMoa1915745

[6] Huang CY, Wang Y, Luo GY, Han F, Li YQ, Zhou ZG, et al. Relationship between PD-L1 expression and CD8^+^ T-cell immune responses in hepatocellular carcinoma. J Immunother. 2017;40(9):323–33. 29028787 10.1097/CJI.0000000000000187

[7] Sun Q, Hong Z, Zhang C, Wang L, Han Z, Ma D. Immune checkpoint therapy for solid tumours: clinical dilemmas and future trends. Signal Transduct Target Ther. 2023;8(1):320. 37635168 10.1038/s41392-023-01522-4 PMC10460796

[8] Hao L, Li S, Deng J, Li N, Yu F, Jiang Z, et al. The current status and future of PD-L1 in liver cancer. Front Immunol. 2023;14:1323581. 38155974 10.3389/fimmu.2023.1323581 PMC10754529

[9] Guo Y, Zhang R, Li W. Emodin in cardiovascular disease: the role and therapeutic potential. Front Pharmacol. 2022;13:1070567. 36618923 10.3389/fphar.2022.1070567 PMC9816479

[10] Sharifi-Rad J, Herrera-Bravo J, Kamiloglu S, Petroni K, Mishra AP, Monserrat-Mesquida M, et al. Recent advances in the therapeutic potential of emodin for human health. Biomed Pharmacother. 2022;154: 113555. 36027610 10.1016/j.biopha.2022.113555

[11] Khan H, Jia W, Yu Z, Zaib T, Feng J, Jiang Y, et al. Emodin succinyl ester inhibits malignant proliferation and migration of hepatocellular carcinoma by suppressing the interaction of AR and EZH2. Biomed Pharmacother. 2020;128: 110244. 32464306 10.1016/j.biopha.2020.110244

[12] Liu Q, Hodge J, Wang J, Wang Y, Wang L, Singh U, et al. Emodin reduces breast cancer lung metastasis by suppressing macrophage-induced breast cancer cell epithelial-mesenchymal transition and cancer stem cell formation. Theranostics. 2020;10(18):8365–81. 32724475 10.7150/thno.45395 PMC7381725

[13] Zhang FY, Li RZ, Xu C, Fan XX, Li JX, Meng WY, et al. Emodin induces apoptosis and suppresses non-small-cell lung cancer growth via downregulation of sPLA2-IIa. Phytomedicine. 2022;95: 153786. 34785104 10.1016/j.phymed.2021.153786

[14] Lim SO, Li CW, Xia W, Cha JH, Chan LC, Wu Y, et al. Deubiquitination and stabilization of PD-L1 by CSN5. Cancer Cell. 2016;30(6):925–39. 27866850 10.1016/j.ccell.2016.10.010 PMC5171205

[15] Yang X, Sun J, Wen B, Wang Y, Zhang M, Chen W, et al. Biejiajian pill promotes the infiltration of CD8^+^T cells in hepatocellular carcinoma by regulating the expression of CCL5. Front Pharmacol. 2021;12: 771046. 34899325 10.3389/fphar.2021.771046 PMC8661106

[16] Wu J, Yuan M, Shen J, Chen Y, Zhang R, Chen X, et al. Effect of modified jianpi yangzheng on regulating content of PKM2 in gastric cancer cells-derived exosomes. Phytomedicine. 2022;103: 154229. 35691076 10.1016/j.phymed.2022.154229

[17] Murakami S, White SM, Mcintosh AT, Nguyen C, Yi C. Spontaneously evolved progenitor niches escape yap oncogene addiction in advanced pancreatic ductal adenocarcinomas. Nat Commun. 2023;14(1):1443. 36922511 10.1038/s41467-023-37147-y PMC10017707

[18] He Z, Hu Y, Zhang Y, Xie J, Niu Z, Yang G, et al. Asiaticoside exerts neuroprotection through targeting NLRP3 inflammasome activation. Phytomedicine. 2024;127: 155494. 38471370 10.1016/j.phymed.2024.155494

[19] Xuan C, Chen D, Zhang S, Li C, Fang Q, Chen D, et al. Isoquercitrin alleviates diabetic nephropathy by inhibiting STAT3 phosphorylation and dimerization. Adv Sci (Weinh). 2025;4: e2414587. 10.1002/advs.202414587 PMC12224983 40184310

[20] Falcinelli M, Al-Hity G, Baron S, Mampay M, Allen MC, Samuels M, et al. Propranolol reduces IFN-γ driven PD-L1 immunosuppression and improves anti-tumour immunity in ovarian cancer. Brain Behav Immun. 2023;110:1–12. 36796704 10.1016/j.bbi.2023.02.011

[21] Gou Q, Dong C, Xu H, Khan B, Jin J, Liu Q, et al. PD-L1 degradation pathway and immunotherapy for cancer. Cell Death Dis. 2020;11(11):955. 33159034 10.1038/s41419-020-03140-2 PMC7648632

[22] Wu M, Xia X, Hu J, Fowlkes NW, Li S. WSX1 act as a tumor suppressor in hepatocellular carcinoma by downregulating neoplastic PD-L1 expression. Nat Commun. 2021;12(1):3500. 34108491 10.1038/s41467-021-23864-9 PMC8190270

[23] Luo S, He J, Huang S, Wang X, Su Y, Li Y, et al. Emodin targeting the colonic metabolism via ppargamma alleviates uc by inhibiting facultative anaerobe. Phytomedicine. 2022;104: 154106. 35728384 10.1016/j.phymed.2022.154106

[24] Saunders IT, Mir H, Kapur N, Singh S. Emodin inhibits colon cancer by altering bcl-2 family proteins and cell survival pathways. Cancer Cell Int. 2019;19:98. 31011292 10.1186/s12935-019-0820-3 PMC6466701

[25] Mcdonald SJ, Vanderveen BN, Velazquez KT, Enos RT, Fairman CM, Cardaci TD, et al. Therapeutic potential of emodin for gastrointestinal cancers. Integr Cancer Ther. 2022;21:1543382605. 10.1177/15347354211067469 PMC8738880 34984952

[26] Sanders B, Ray AM, Goldberg S, Clark T, Mcdaniel HR, Atlas SE, et al. Anti-cancer effects of aloe-emodin: a systematic review. J Clin Transl Res. 2018;3(3):283–96. 30895270 PMC6426255

[27] Tuminello S, Sikavi D, Veluswamy R, Gamarra C, Lieberman-Cribbin W, Flores R, et al. PD-L1 as a prognostic biomarker in surgically resectable non-small cell lung cancer: a meta-analysis. Transl Lung Cancer Res. 2020;9(4):1343–60. 32953509 10.21037/tlcr-19-638 PMC7481631

[28] Xu X, Xie T, Zhou M, Sun Y, Wang F, Tian Y, et al. Hsc70 promotes anti-tumor immunity by targeting PD-L1 for lysosomal degradation. Nat Commun. 2024;15(1):4237. 38762492 10.1038/s41467-024-48597-3 PMC11102475

[29] Lemma EY, Letian A, Altorki NK, Mcgraw TE. Regulation of PD-L1 trafficking from synthesis to degradation. Cancer Immunol Res. 2023;11(7):866–74. 37290119 10.1158/2326-6066.CIR-22-0953 PMC10320477

[30] Zhu L, Kuang X, Zhang G, Liang L, Liu D, Hu B, et al. Albendazole induces immunotherapy response by facilitating ubiquitin-mediated PD-L1 degradation. J Immunother Cancer. 2022;10(5): e003819. 35577504 10.1136/jitc-2021-003819 PMC9115032

[31] Wu Y, Zhang C, Liu X, He Z, Shan B, Zeng Q, et al. ARIH1 signaling promotes anti-tumor immunity by targeting PD-L1 for proteasomal degradation. Nat Commun. 2021;12(1):2346. 33879767 10.1038/s41467-021-22467-8 PMC8058344

[32] Zhang N, Liu X, Liu L, Deng Z, Zeng Q, Pang W, et al. Glycogen synthase kinase-3β inhibition promotes lysosome-dependent degradation of c-FLIPL in hepatocellular carcinoma. Cell Death Dis. 2018;9(2):230. 29445085 10.1038/s41419-018-0309-3 PMC5833564

[33] Cervello M, Augello G, Cusimano A, Emma MR, Balasus D, Azzolina A, et al. Pivotal roles of glycogen synthase-3 in hepatocellular carcinoma. Adv Biol Regul. 2017;65:59–76. 28619606 10.1016/j.jbior.2017.06.002

[34] Fang G, Zhang P, Liu J, Zhang X, Zhu X, Li R, et al. Inhibition of GSK-3β activity suppresses hcc malignant phenotype by inhibiting glycolysis via activating AMPK/mTOR signaling. Cancer Lett. 2019;463:11–26. 31404613 10.1016/j.canlet.2019.08.003

[35] Chen W, Yang X, Sun J, Chen Y, Zhao W, He C, et al. Biejiajian pill inhibits progression of hepatocellular carcinoma by downregulating PDGFRβ signaling in cancer-associated fibroblasts. J Ethnopharmacol. 2023;301: 115825. 36240978 10.1016/j.jep.2022.115825

[36] Sun J, Yang X, Sun H, Huang S, An H, Xu W, et al. Baicalin inhibits hepatocellular carcinoma cell growth and metastasis by suppressing ROCK1 signaling. Phytother Res. 2023;37(9):4117–32. 37246830 10.1002/ptr.7873

[37] Li CW, Lim SO, Xia W, Lee HH, Chan LC, Kuo CW, et al. Glycosylation and stabilization of programmed death ligand-1 suppresses T-cell activity. Nat Commun. 2016;7:12632. 27572267 10.1038/ncomms12632 PMC5013604

[38] Qin B, Zeng Z, Xu J, Shangwen J, Ye ZJ, Wang S, et al. Emodin inhibits invasion and migration of hepatocellular carcinoma cells via regulating autophagy-mediated degradation of snail and β-catenin. BMC Cancer. 2022;22(1):671. 35715752 10.1186/s12885-022-09684-0 PMC9206273

[39] Raskov H, Orhan A, Christensen JP, Gogenur I. Cytotoxic CD8^+^ T cells in cancer and cancer immunotherapy. Br J Cancer. 2021;124(2):359–67. 32929195 10.1038/s41416-020-01048-4 PMC7853123

[40] Chen Y, Yu D, Qian H, Shi Y, Tao Z. CD8^+^ T cell-based cancer immunotherapy. J Transl Med. 2024;22(1):394. 38685033 10.1186/s12967-024-05134-6 PMC11057112

[41] Ai L, Chen J, Yan H, He Q, Luo P, Xu Z, et al. Research status and outlook of PD-1/PD-L1 inhibitors for cancer therapy. Drug Des Devel Ther. 2020;14:3625–49. 32982171 10.2147/DDDT.S267433 PMC7490077

