# Supplementary material for: Emodin promotes GSK-3β-mediated PD-L1 proteasomal degradation and enhances anti-tumor immunity in hepatocellular carcinoma
Source: Chin Med. 2025 Aug 13;20:126. doi: 10.1186/s13020-025-01146-6 (PMC12345107; doi:10.1186/s13020-025-01146-6)

**Supplementary materials**

**Supplementary Figure1 | The effect of emodin on **PD-L1 ubiquitination levels.** Immunoprecipitation (IP) with anti-PD-L1 antibody followed by immunoblotting (IB) with anti-ubiquitin antibody. Representative blots from three independent experiments are shown.**


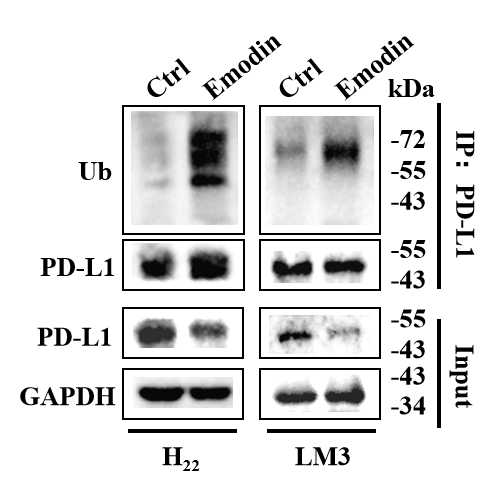


**Supplementary Figure 2 | Combination treatment between emodin with anti-PD-1 *in vivo*.** (A) Body weight. (B) Images of typical tumors. (C) Tumor size was recorded at specified times. (D) Weight of the tumors. Data are from three independent experiments (mean ± SD, **P* < 0.05, ***P* < 0.01 vs. controls. ^#^*P* < 0.05 vs. Anti-PD-1 group).


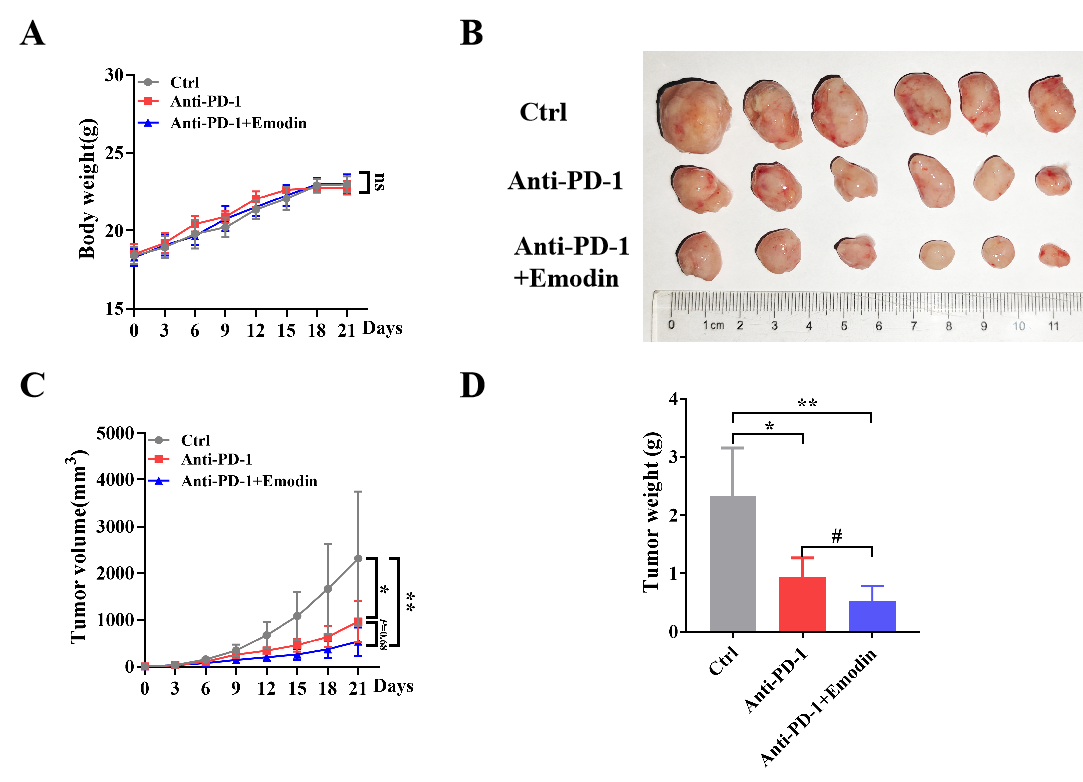

Supplement: Supplementary file 1 [file 13020_2025_1146_MOESM1_ESM.docx]
